# The Role of Sustained Type I Interferon Secretion in Chronic HIV Pathogenicity: Implications for Viral Persistence, Immune Activation, and Immunometabolism

**DOI:** 10.3390/v17020139

**Published:** 2025-01-22

**Authors:** Eman Teer, Nyasha C. Mukonowenzou, M. Faadiel Essop

**Affiliations:** 1 Centre for Cardio-Metabolic Research in Africa, Department of Physiological Sciences, Stellenbosch University, Stellenbosch 7600, South Africa; eman.ab.teer@gmail.com (E.T.); 25707264@sun.ac.za (N.C.M.); 2 Centre for Cardio-Metabolic Research in Africa, Division of Medical Physiology, Faculty of Medicine and Health Sciences, Stellenbosch University, Cape Town 8000, South Africa

**Keywords:** Type I interferons (IFN-I), IFN-stimulated genes (ISGs), HIV-pathogenicity, acute and chronic stages of HIV infection, immune activation, immunometabolism

## Abstract

Human immunodeficiency virus (HIV) infection induces chronic immune activation by stimulating both the innate and adaptive immune systems, resulting in persistent inflammation and immune cell exhaustion. Of note, the modulation of cytokine production and its release can significantly influence the immune response. Type I interferons (IFN-Is) are cytokines that play a crucial role in innate immunity due to their potent antiviral effects, regulation of IFN-stimulated genes essential for viral clearance, and the initiation of both innate and adaptive immune responses. Thus, an understanding of the dual role of IFN-I (protective versus harmful) during HIV-1 infections and elucidating its contributions to HIV pathogenesis is crucial for advancing HIV therapeutic interventions. This review therefore delves into the intricate involvement of IFN-I in both the acute and chronic phases of HIV infection and emphasizes its impact on viral persistence, immune activation, and immunometabolism in treated HIV-infected individuals.

## 1. Introduction

Cytokines are a broad group of signaling proteins that are rapidly produced after cellular activation. They act as humoral regulators that can modulate cellular function and also govern developmental and pathological conditions [1]. Type I interferons (IFN-Is) are important cytokines that play a key role in fighting infections and pathogen clearance as they directly inhibit viral replication in infected cells and enhance innate immunity through the modulation of various cell types [2]. They can elicit diverse effects (direct and indirect) on numerous target cell types during a viral infection(s) as most human cells express IFN-I receptors. For example, they can promote host defenses by inducing antiviral effector molecules encoded by IFN-stimulated genes (ISGs) and thereby enhance the effector function of several immune modulators including dendritic, B-, and T-cells [3]. Moreover, they are central to driving antiviral immune responses by inhibiting viral replication and boosting antigen presentation during the early innate phase [4]. They can also trigger adaptive immune responses through direct and indirect actions on T- and B-cells involved in the memory response [5]. Thus, IFN-Is function as master regulators with their relatively early induction during a viral infection leading to the modulation of downstream signaling cascades [6]. This can result in the promotion of both pro- and anti-inflammatory responses (based on the underlying physiologic context) and enhances the effector function of immune cells for the successful resolution of infections [5,6].

However, the functional roles of IFN-Is under conditions of sustained secretion are not fully understood. Emerging research suggests that they may contribute to host-driven immunopathology during chronic infection(s) [7,8]. Persistent IFN-I signaling is implicated in chronic immune activation, immune exhaustion, and the establishment of latent reservoirs that allow human immunodeficiency virus (HIV) to evade clearance and persist in the host [8]. This review therefore aims to address this knowledge gap by focusing on the functional roles of IFN-Is in the context of HIV infection but will adopt a more holistic approach. Thus, we initially focus on characterizing IFN-Is protective roles during the acute stages of infection and thereafter shift the spotlight to its detrimental effects during chronic HIV infection (with treatment). We also explore how these immunomodulatory cytokines can contribute to HIV pathogenicity and thereafter reflect on the prospects of exploiting such knowledge for therapeutic purposes. Lastly, we will outline how changes in metabolic pathways affect both immune function and tissue/organ physiology and briefly discuss the relationship between IFN-I and immunometabolism.

## 2. HIV Infection and Innate Immunity Sensing/Signaling

Protective immunity against microbes can be mediated by both early and late responses of the innate and adaptive immune systems. Although the innate immune response is non-specific, it ensures a rapid defense against invading microbes during the first few hours/days after infection [9]. It is constituted of several important components that include effector cells, antimicrobial peptides, soluble mediators, cell receptors, and physical and anatomical barriers [10]. Moreover, the mononuclear phagocyte system (originating from myeloid progenitors in bone marrow) that comprises monocytes, macrophages, and dendritic cells represents the foundation of the innate immune response [11].

Throughout HIV-acquired immunodeficiency syndrome (AIDS), early events after HIV-1 infection affect the course of viral replication and disease progression during the acute stages of the infection [12,13]. HIV ensures its survival by replication and reproduction in the infected cell via uncoating, nucleic acid replication, translation into viral proteins, and incorporation into new virions [14]. However, attenuated viral replication during an acute HIV infection often occurs before the induction of adaptive immunity (e.g., CD8+ T-cells) responses [12]. This indicates that, in addition to its role in activating adaptive immunity, the innate immune system’s early responses are crucial for antiviral control [15].

Unlike the adaptive immune system that uses antigen-specific receptors to recognize foreign antigens, the innate immune response can trigger processes of pathogen sensing and intracellular signaling pathways [16]. Here, innate immune system sensing employs a pattern recognition receptor (PRR) to detect molecular patterns associated with viruses [12]. PRR ligands belong to three major categories depending on their origin, i.e., pathogen-associated molecular patterns (PAMPs), damage-associated molecular patterns (DAMPs) and aryl hydrocarbon receptor ligands (AhR ligands) [17]. PRRs detect viral components such as genomic deoxyribonucleic acid (DNA), single-stranded ribonucleic acid (RNA), double-stranded RNA, RNA with 5′-triphosphate ends, and viral proteins [18].

During acute HIV-1 infection, viral PAMPs are recognized by the host cell’s PRRs which then activates a signaling cascade that initiates innate intracellular antiviral responses [19]. This cell-intrinsic response aims to restrict viral replication and its further spread [12,20]. The HIV-1 infection also triggers innate immune receptors, including Toll-like receptor 7 (TLR7) and TLR8 [19]. Furthermore, cytoplasmic cyclic receptors including cyclic guanosine monophosphate-adenosine monophosphate synthase (cGAS) and interferon γ-inducible protein 16 (IFI16) can sense HIV complementary DNA (cDNA) [21,22]. This leads to the activation of intracellular signal transduction pathways involving interferon regulatory transcription factor 3 and nuclear factor kappa B (NF-κB) [23]. This results in the robust activation of dendritic cells and the release of copious amounts of IFN-I and other cytokines that help to limit viral replication within infected cells.

**Figure 1:**
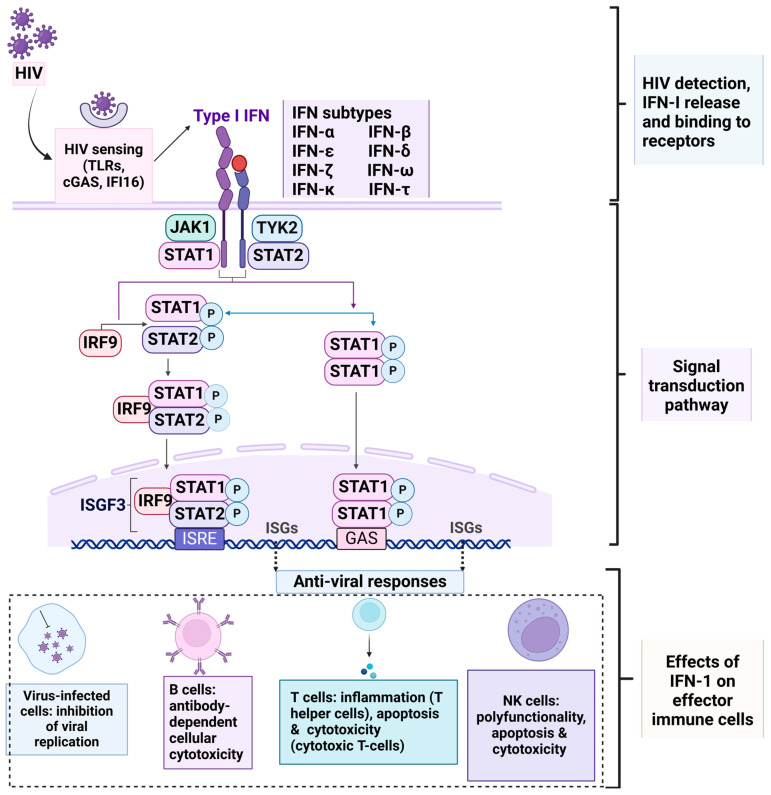
General IFN-I signaling cascade and downstream immune cell responses in HIV infection. HIV sensing and signaling by various cells (primarily plasma dendritic cells) result in protein factors’ recruitment, activation, and transcription. This triggers the release of IFN-I which induces the expression of ISGs via the JAK/STAT signal transduction pathway. Downstream effector immune cells including natural killer cells, B-, and T-cells are responsible for a diverse range of antiviral activity. HIV: human immunodeficiency virus; TLRs: Toll-like receptors; cGAS: cyclic GMP-AMP synthase; IFI16: interferon-inducible protein 16; JAK: Janus kinase; STAT: signal transducers and activators of transcription; IRF9: interferon regulatory transcription factor 9; ISRE: interferon-sensitive response element; ISGs: interferon-stimulated genes; ISGF3: IFN-stimulated gene factor 3; NK cells: natural killer cells; P: phosphorylation. Created in BioRender https://biorender.com/.

Interferons are classified into three groups based on the structure of their receptors, namely Type I (IFN-α, IFN-β, IFN-κ, IFN-δ, IFN-ε, IFN-τ, IFN-ω, and IFN-ζ), Type II (IFN-γ), and Type III (IFN-λ1, IFN-λ2, and IFN-λ3). Here, IFN-I binds to a cell surface receptor complex known as the IFN-α/β receptor which consists of IFN-α/β receptor-1 and IFN-α/β receptor-2 chains [24]. Dendritic cells act as effectors of the innate immune system through the production of IFN-I and also by activation of ISGs via the Janus kinase/signal transducers and activators of transcription (JAK/STAT) pathways (Figure 1) [21,23,25]. Several ISGs code for proteins that play diverse functional roles, e.g., the induction of apoptosis and the inhibition of growth via restriction factors [26]. Such antiviral activities can affect every stage of the HIV life cycle, i.e., from its binding to cell surface receptors, maturation, and virion release as outlined in Table 1 [27].

IFN-I are key contributors to effective antiviral responses in the context of acute viral infection and are important regulators of innate and adaptive immune responses (via direct and indirect mechanisms) [4]. The induction of IFN-I during viral infections occurs via two major pathways, firstly through retinoic acid-inducible gene-I-like receptors that are cytoplasmic sensors that recognize viral double-stranded RNA in infected cells [28]. Secondly, through TLR-3, -7, and -9 that are localized in the endosomal compartment of specialized cells such as dendritic cells and recognize double-stranded DNA, single-stranded RNA and unmethylated CpG-rich DNA. Upon such activation, IFN-Is play a broad protective role by suppressing viral replication, inducing inflammatory pathways, regulating cell survival mechanisms, and promoting maturation and differentiation of antigen-presenting cells such as dendritic cells and monocytes/macrophages [29,30]. IFN-Is also play an important role in the development of T-cell-mediated antigen-specific immune responses by stimulating antigen-presentation [31].

The innate immune antiviral response also includes the secretion of cytokines and chemokines that activate innate immune cells such as macrophages, natural killer (NK), and dendritic cells, and attracts them to the sites of infection and local lymphatic tissues. The mobilization of antiviral innate effector cells subsequently contributes to decreased viremia and also the type of adaptive immune response to an HIV-1 infection [20]. Numerous studies also investigated and compared the role of innate immunity in elite controllers who are classified as HIV-infected long-term non-progressors with natural progressors [32,33,34]. Elite controllers exhibit a strong innate immune response with macrophages, NK and dendritic cells, mediating highly effective phagocytosis that leads to the death of infected cells. In parallel, there is also a concomitant rise in the secretion of inflammatory cytokines such as IFN-I (IFN-α, IFN-β), tumor necrosis factor-alpha (TNF-α), and interleukin (IL)-12 [35]. During the early stages of HIV-1 infection, the acute cytokine cascade is significantly enhanced when compared to other viral infections such as hepatitis B and C infections [36]. Thus, while the presence of such immunomodulators may be part of a robust immune response against the incoming pathogen(s), its intensity and magnitude may also contribute (in part) to the observed immunopathology associated with early HIV disease status [36].

**Table 1:** Restriction factors in HIV infection.

Restriction Factor	HIV Antiviral Mechanism	References
Interferon-induced transmembrane (IFITM)	Broad spectrum of actions ranging from restricting viral entry to inhibiting viral protein synthesis. ○ IFITM1 inhibits production of the viral protein gag; ○ IFITM 3 prevents the cleavage of the viral protein env; ○ IFITMδ20 preferentially restricts entry of the C-X-C receptor 4 (CXCR4) HIV variant.	[37,38,39]
Apolipoprotein B mRNA editing enzyme catalytic polypeptide like-3 (APOBEC3)	Restrict reverse transcription of HIV RNA. ○ APOBEC3G causes hypermutation during HIV reverse transcription making the proviruses incapable of replication; ○ APOBEC3A specifically inhibits HIV replication in myeloid cells; ○ In viral protein vif-deficient HIV, APOBEC3D, APOBEC3F, APOBEC3G, and APOBEC3H prevent viral replication.	[40,41]
Myxovirus resistance-2 (MX2)	Inhibits translocation of the HIV cDNA post-entry thereby preventing proviral integration.	[42,43]
ISG15	Prevents ubiquitination of the viral protein gag, thereby inhibiting HIV replication and the assembly and release of virions.	[44,45]
Tripartite motif-containing protein-5 (TRIM5α)	Detects HIV capsids and causes their disassembly thereby inhibiting HIV replication.	[46]
SAM and HD domain containing protein 1 (SAMHD1)	Decreases the availability of deoxynucleotide triphosphates available for viral replication, particularly in macrophages.	[47,48]
Serine incorporator (SERINC-3/5)	Reduces HIV infectivity ○ SERINC5 prevents the fusion of HIV and host cells via inactivation of env and structural changes to gp41; ○ SERINC3 and SERINC5 limit the spread of HIV to new target cells by decreasing the fusion of replicated (offspring) env proteins and host cells	[49,50]
Guanylate binding protein-2/5 (GBP2/GBP5)	Attenuates HIV infectivity ○ GBP5 impairs env virion processing and incorporation thereby reducing HIV infectivity; ○ GBP2 and GBP5 decrease furin activity. Furin is a convertase enzyme necessary for the conversion of gp 160 (env precursor) to functional gp 120 and gp 41.	[51,52]
Endoplasmic reticulum α1,2-mannosidase-I (ERManI)	Degrades the viral envelope thereby inhibiting HIV replication. ○ Degradation occurs through the ER-associated protein degradation (ERAD) pathway.	[53]
Zinc-finger antiviral protein (ZAP)	Targets viral RNA ○ Binds to and degrades viral mRNA thereby preventing translation and viral replication.	[54]
Bone marrow stromal cell antigen-2 (BST2)/Tetherin	Prevents new virions from budding and concomitantly promotes NF-κB signaling.	[55]
Schlafen 11 (SLFN 11)	Binds to transfer RNA and inhibits translation of viral proteins by altering codon availability and usage.	[56]
Tryptophan-rich sensory protein (TSPO)	Promotes the degradation of env through the ERAD pathway.	[57]

## 3. IFN-I Responses During an HIV Infection

The IFN-I response is robust, well-regulated, and can result in the resolution of many viral infections, e.g., an acute HIV infection. Moreover, treatments based on early IFN-I administration can improve immunopathology and are relatively effective in blunting infections such as those mediated by the hepatitis C virus, and also other health complications like multiple sclerosis and non-Hodgkin’s lymphoma [58,59]. However, IFN signaling can also lead to paradoxical outcomes during an HIV infection (even if individuals are receiving antiretroviral therapy [ART]). While eliciting antiviral effects as described, it can also contribute to persistent immune activation [60]. This intrigued researchers to pursue several questions such as: (1) why do the IFN-I signaling pathways fail to clear the HIV-1 during the acute stage and/or what is its role during chronic HIV infection? (2) is ART administration during the acute and/or chronic stages of HIV infection indeed beneficial? and (3) does IFN-I supplementation during acute and/or chronic infections improve antiviral activity while attenuating immune activation effects? Although such vexing questions remain the focus of ongoing investigations in the field, we pose a unique question for this review article, i.e., would there be any potential crosstalk between IFN-I and immunometabolism and if so, what type of functional outcomes would manifest during an HIV infection?

## 4. Role of IFN-I During Acute Stages of HIV Infection

The importance of IFN-I in attenuating viral replication during the acute stages of HIV is well established by in vitro and in vivo studies [61]. For example, administration of an IFN-I receptor antagonist in an acute simian immunodeficiency virus (SIV) rhesus macaque model resulted in increased viral loads, the onset of AIDS, and death of the animals within 30 weeks [62]. Moreover, untreated rhesus macaques displayed lower viral loads and survived the entire 44-week study period [62]. The administration of IFN-I before infection and for up to 4 weeks post-infection also resulted in decreased transmission rates in the same study. Such research reveals the significance of timing in terms of therapeutic interventions to enhance HIV antiviral activities. The importance of IFN-I is further strengthened by others showing that HIV-infected individuals exhibited an intense cytokine storm with increased plasma IFN-I levels during the acute stages of the infection [36]. In support, humanized cells [63] exhibited a more dominant IFN-α14 response (versus IFN-α2) thereby highlighting the ubiquity of the IFN-I response in terms of controlling the HIV infection [64]. Thus, timing, duration, study type (in vivo/in vitro), and IFN-I subtypes are all important parameters to consider when tackling such studies.

HIV-infected cell lines are increasingly used to investigate the role of IFN-I during acute HIV infection and findings show that such cells displayed increased susceptibility to IFN-I [65,66,67]. Interestingly, while the acute phase of HIV infection shows increased secretion and expression of IFN-I and ISGs in rhesus macaques [62], host–virus interactions limit ISG functionality thereby dampening the IFN response. Viral accessory proteins such as nef and vif subdue ISGs thereby inhibiting downstream effector functions, counteracting host restriction factors, and degrading transcription factors via lysosomal and/or ubiquitin pathways [68,69]. The virus can evade IFN-I regulation, thereby contributing to viral persistence and the establishment of latent reservoirs. The differences observed between in vitro and in vivo models could be due to the provision of partial information regarding the interrelated immune signaling pathways involved in infected cells [70,71]. Furthermore, heterogeneity among cell lines in their responses to HIV infection and IFN-signaling was observed and may contribute to observed differences [71].

As these data revealed, IFN-I immunotherapy offered initial promise and was thus considered as a possible treatment early-on during the HIV pandemic. However, as subsequent research work showed its limitations in this context, the focus then shifted to a combination of IFN-I therapy and ART. Here, it was reasoned that adding IFN-I to combination antiretroviral therapy (cART) during the acute stages of HIV infection would decrease viral reservoirs and more rapidly slow disease progression versus sole cART administration. In support, a preclinical mouse study demonstrated that concurrent administration of IFN-α14 (potent IFN-I subtype) and ART caused a greater reduction in plasma viremia versus sole ART administration [72]. However, this treatment failed to lower latent viral reservoirs. Several other studies corroborated such findings with mixed outcomes, i.e., some benefits versus sole ART administration, but also ineffective as an HIV cure [61].

## 5. Role of IFN-I During Chronic Stages of HIV Infection

Although IFN-I are robustly upregulated in natural SIV hosts and non-natural ones during the acute stages of an HIV infection, such responses differ during the chronic stages. Here, natural hosts downregulate IFN-I responses during the early chronic stages, while such responses persist and lead to deleterious chronic immune activation in non-natural hosts [73]. In support, models of SIV infection showed that long-time non-progression macaques can induce rapid and transient IFN-I high levels that then decline during the chronic stages [61]. Moreover, in vitro [74,75] studies using plasma dendritic cells demonstrated that HIV can mask its recognition by cyclic GMP-AMP synthase (cGAS) and IFI16 via degradation of their substrate cDNA [76]. Such IFN-I-mediated persistent immune activation through amplifying IFN-I signaling can lead to ISG upregulation and can elicit both antiviral and immunomodulatory effects. The ISGs can also induce the expression of cytosolic nucleic acid sensors like cGAS which can detect HIV DNA and trigger further IFN-I production. This creates a self-reinforcing loop where IFN-I drives ISG expression, ISGs activate nucleic acid sensors, and the sensors induce more IFN-I release. Such sustained IFN-I signaling and ISG expression can thus fuel chronic immune activation and inflammation characteristic of progressive HIV. Although not fully elucidated, other mechanisms that contribute to such chronic HIV infection include thymic dysfunction due to chronic immune activation, increased apoptosis of infected and bystander cells, delayed immune recovery, enhanced T-cell exhaustion, and immune dysregulation [61,77]. In agreement, a strong correlation was reported between IFN-I levels and HIV disease progression [78].

How do ART regimens influence such IFN-I-mediated effects during an HIV infection? Here, the findings reveal mixed outcomes with some persons living with HIV (and on treatment) still displaying signatures of persistent immune activation and low-grade inflammation with continuous IFN-I signaling [60]. Moreover, others found that circulating IFN-I levels correlated inversely with CD4 T-cell count, positively with plasma HIV-1 RNA levels and CD38 expression on CD8 T-cells [78]. In agreement, inhibition of IFN-I signaling in HIV-infected humanized mice decreased immune activation and HIV reservoirs, while slowing viral rebound after ART cessation [79]. This could potentially reduce end-stage pathology such as cardiovascular, renal, and neural complications that can present in HIV-infected individuals. These results demonstrate the role of IFN-I as a significant contributor to immune activation during an HIV infection. However, others found that although the inhibition of IFN-I signaling lowered downstream-related inflammatory pathways (e.g., ISG downregulation), this did not result in a decrease in plasma viremia and immune activation [80]. Clinical trials targeting the inhibition of IFN-I signaling (using natural, recombinant, and pegylated IFN) also did not yield the same benefits as shown by in vitro and in vivo studies [28]. The reasons for such differences should be further investigated if IFN-I is to be considered as a therapeutic modality in the context of HIV patient management. Moreover, biological sex is an important factor to consider in terms of the role of IFN-I signaling during chronic HIV infection as females exhibit a more robust immune response and enhanced expression of ISGs. Several studies [81,82,83,84] found that females produce higher amounts of IFN-I upon TLR-7 activation compared to males. This seems to be due to differences in both sex-chromosomal and sex-steroid hormone-mediated signaling for TLR-7 and plasma dendritic cells [85]. Regarding sex-steroid hormone-mediated signaling, both male and female sex hormone receptors are expressed by immune cells and therefore play an immunomodulatory role [86]. Estrogen, a dominant female hormone, exhibits an immune-stimulatory effect while testosterone, a dominant male hormone, exhibits an immune-suppressive effect [87]. Estrogen receptors regulate gene transcription directly, e.g., via the transcription of IRF5 or NF-κB, thereby contributing to the increased IFN-I production by plasma dendritic cells [82]. Concerning sex-chromosomal differences, females contain two X chromosomes which encode more genes than the Y chromosome and such genes contain immunomodulatory molecules (e.g., TLR-7 and micro-RNAs) which impact IFN-I responses [88,89]. These combined effects of biological sex on IFN-I responses ultimately impact effector cell (e.g., NK and CD8+) responses and contribute to the observed differences in immune responses in individuals of different sex [90]. Thus, we propose that future studies should consider various factors such as sex, timing, duration, IFN-I subtype, and concurrent ART administration.

### 5.1. Role of Interferons and Trained Innate Immune Responses in Elite Controllers

IFN-I, particularly IFN-α and IFN-β, play a pivotal role in antiviral immunity and are central to the phenomenon of elite control during HIV infection. Elite controllers, a rare subset of individuals who naturally suppress HIV without antiretroviral therapy, exhibit a highly regulated IFN-I response that is critical to their ability to control the virus [91,92]. Upon HIV infection, IFN-Is are rapidly produced, inducing the expression of ISGs that inhibit viral replication. In elite controllers, this response is finely balanced, achieving effective viral suppression without triggering the chronic immune activation that is typically observed in individuals who progress to AIDS [3]. This balance may be attributed to selective activation of specific ISGs (e.g., ISG15) that mediate antiviral effects while avoiding the immune exhaustion associated with prolonged IFN signaling [77]. Additionally, IFN-α can enhance the ability of HIV-1-specific CD8+ T-cells from elite controllers to suppress HIV-1 replication in infected CD4+ T-cells, suggesting a functional link between innate immune modulators and adaptive CD8+ T-cell responses [93].

Beyond their direct antiviral effects, IFN-Is also augment the activity of both innate and adaptive immune cells. These cytokines enhance the cytotoxicity of NK cells and improve antigen presentation to CD8+ T-cells by upregulating major histocompatibility complex class I molecules [93,94]. Furthermore, IFN-Is contribute to shaping trained innate immunity, a process through which innate immune cells such as monocytes and macrophages develop a memory-like capacity to mount stronger responses to repeated encounters with HIV [60]. This phenomenon involves epigenetic reprogramming and metabolic rewiring, enabling these cells to function more effectively. For example, monocytes in elite controllers exhibit increased resistance to HIV infection, while NK cells display heightened cytotoxic activity due to IFN-I-driven priming [95].

The interaction between IFN-I and trained innate immunity is a critical factor in the immune control observed in elite controllers [60]. IFN-Is act not only as initial training stimuli but also as regulators that fine-tune immune responses, enabling viral suppression without excessive inflammation. This delicate interplay highlights the therapeutic potential of targeting IFN-I pathways or inducing trained immunity to replicate the immune mechanisms observed in elite controllers [96]. Gaining a deeper understanding of these mechanisms may pave the way for innovative strategies to manage HIV and other chronic infections, offering hope for more effective therapies and improved outcomes.

### 5.2. IFN-I: Persistent HIV and Viral Reservoirs

IFN-I, including IFN-α and IFN-β, play a dual role in HIV infection. While they provide critical antiviral benefits during the acute phase of infection, their prolonged signaling in the chronic phase contributes significantly to viral persistence [3]. Persistent IFN-I signaling drives chronic immune activation, a hallmark of HIV infection, which creates an inflammatory environment that perpetuates immune dysfunction [97]. Chronically activated CD4+ T-cells, which are more susceptible to HIV infection, act as a continuous source of viral replication. This ongoing activation not only damages tissues and disrupts immune homeostasis but also impairs the immune system’s capacity to clear infected cells, allowing the virus to persist [8].

Prolonged IFN-I signaling also contributes to immune exhaustion, particularly in CD8+ T-cells, that is vital for HIV control [98]. Metabolic reprogramming in these cells, induced by IFN-I, leads to increased expression of inhibitory receptors such as programmed cell death protein 1 (PD-1) and cytotoxic T-lymphocyte-associated protein 4 (CTLA-4), reduced proliferation, and impaired effector function. These changes diminish their ability to effectively suppress viral reservoirs [98].

Furthermore, IFN-I signaling disrupts innate immune responses by impairing the functions of dendritic cells, macrophages, and NK cells. This dysregulation weakens the immune system’s ability to eliminate infected cells and further supports HIV persistence [6]. In the gut mucosa, IFN-I exacerbates tissue damage, promoting microbial translocation and systemic inflammation, which can sustain viral replication and reservoir formation [99].

Metabolic reprogramming driven by IFN-I further contributes to HIV persistence. While these metabolic changes initially provide the energy required for antiviral responses, prolonged IFN-I activity leads to mitochondrial dysfunction, resource depletion, and exhaustion of T-cells and other immune cells [100]. This metabolic stress compromises immune functionality and fosters an environment that favors viral survival and replication [101].

IFN-Is play a dual role in reservoir dynamics, i.e., while they contribute to the establishment and maintenance of viral latency by creating an antiviral state that suppresses active viral replication, they can also activate latent reservoirs under certain conditions, making them susceptible to immune clearance [102]. These opposing effects highlight the intricate relationship between IFN-I signaling and HIV persistence. Recent studies suggest that IFN-Is may upregulate the expression of latency-reversing agents or trigger immune responses capable of targeting reactivated reservoirs [103]. However, chronic IFN-I signaling can also lead to immune exhaustion and inflammation, impairing the ability of immune cells to effectively clear these reservoirs [25].

## 6. IFN-I: Persistent Immune Activation and Metabolic Reprogramming During HIV Infection

While the importance of peripheral and tissue-specific immune responses in viral control is undisputed, emerging evidence suggests that metabolic pathways also play a key role in immune cell function [104]. The induction of innate immune responses requires significant metabolic resources, including energy, enzymes, and intermediates of macromolecular biosynthesis, such as transcription and translation [105]. Thus, several interrelated processes including aerobic glycolysis, the tricarboxylic acid cycle, the pentose phosphate pathway, fatty acid β-oxidation, fatty acid synthesis, oxidative phosphorylation, and amino acid synthesis (particularly glutaminolysis) can all influence and regulate immune function [106].

A plethora of immune cell phenotypes are associated with distinct intracellular metabolic pathway signatures that can be reprogrammed under various physiological and/or pathological conditions [107]. The study of the interplay between immune cell activity and metabolism (termed immunometabolism) has garnered research interest in recent years and is divided into cellular immunometabolism and tissue immunometabolism [108]. Cellular immunometabolism refers to metabolic reprogramming that alters immune cell activities while tissue immunometabolism refers to the effects of reprogrammed cells on tissues and/or organs [109]. Immunometabolism has been implicated in HIV pathogenesis as the virus can utilize various metabolic pathways to enable its survival advantage, and to inactivate immune responses to achieve maximal reproduction and/or persistence [106].

It is widely accepted that HIV readily infects activated cells, while strongly resisted by quiescent and refracted by naïve cells [110,111]. However, most of the research done in the immunometabolism and HIV field focused on macrophages and T-cells. Here, oxidative phosphorylation is the predominant mode of metabolism during the resting or quiescent states, using amino acids, lipids, and pyruvate as fuel substrates. However, upon immune cell activation, there is an upregulation of glucose and amino acid transporters leading to the predominance of aerobic glycolysis and glutaminolysis [108,112]. Once such fuel substrates are increasingly utilized by these immune cells, this can give rise to diverse metabolic substrates that are permissive to HIV (at every life cycle stage) to facilitate the replication, maturation, and release of productive viruses (reviewed in [106]).

Interferons and ISGs exert profound effects on cellular metabolism and recent evidence suggests that antiviral responses are supported by an IFN-induced metabolic re-programming [113]. While metabolic pathways regulating IFN-I secretion by dendritic cells in the context of HIV infection largely remained unexplored, early findings from non-HIV-infected cells are concordant with data generated by macrophage and T-cell research studies [114,115]. However, emerging findings suggest that dendritic cells display a preference for fatty acid β-oxidation and oxidative phosphorylation [116,117]. In support, some found that fatty acid β-oxidation blunted dendritic cell activation [101]. These findings were corroborated in a recent comprehensive study [118] to thereby challenge earlier findings implicating the predominance of aerobic glycolysis in terms of dendritic cell function. Here, the authors indicated that all three pathways are essential for dendritic cell survival and IFN secretion. Of note, pyruvate is crucial for the activation of AMP-activated protein kinase which plays a key role in regulating the process of dendritic cell activation. The use of well-defined TLRs and culturing methods that coincide with IFN secretion are some of the differences between this study [118] and previous work [114,117].

In T-cells, sustained IFN-I signaling during chronic HIV infection drives metabolic shifts that contribute to immune exhaustion [98]. These shifts include increased glycolysis, mitochondrial dysfunction, and the accumulation of reactive oxygen species, which collectively impair T-cell proliferation and effector function [98]. T-cell exhaustion is characterized by reduced functionality, upregulation of inhibitory receptors such as PD-1, and an inability to sustain robust antiviral responses [119]. The persistent metabolic dysregulation caused by prolonged IFN exposure compromises the bioenergetic capacity of T-cells, further perpetuating immune dysfunction [120].

**Figure 2:**
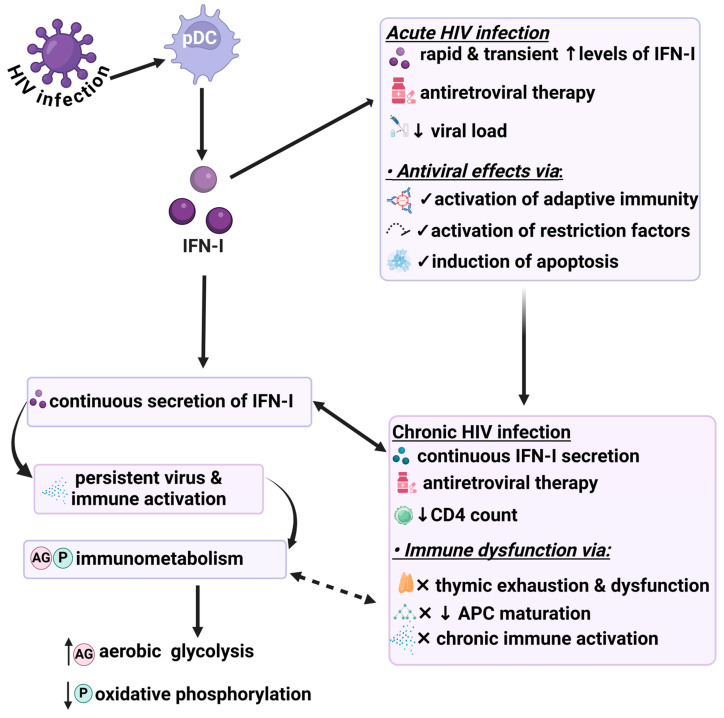
Acute and chronic effects of IFN-I and possible effects of immunometabolism during HIV infection. Despite the beneficial role of IFN-I secretion in acute HIV infection, sustained IFN-I secretion during HIV infection leads to a persistent viral phenotype and immune activation. Furthermore, it indirectly promotes alterations in immunometabolism which may fuel immune dysfunction. APC: antigen-presenting cells; HIV: human immunodeficiency virus; pDCs: plasma dendritic cells; and IFN-I: interferon-1. Created in BioRender https://biorender.com/.

More studies involving HIV-infected cells, animal models, and clinical work are required to better understand the challenges and potential benefits of targeting immunometabolic pathways in terms of IFN-I signaling. Thus, we propose that cellular metabolism of immune cells is an attractive target to develop improved diagnostic tools and novel therapeutic strategies in the context of interactions between HIV and its host. Sustained secretion of IFN-I drives viral persistence and immune activation, while there is strong evidence that this can lead to immune dysregulation as a primary driver of HIV pathogenicity. Of note, recent studies indicate that metabolic reprogramming of immune cell subsets is linked to such immunological activation and dysfunction [121]. Thus, we propose that sustained IFN-I secretion indirectly leads to immunometabolic changes during HIV infection (Figure 2).

### 6.1. IFN-I: Interventional Studies in Non-Human Primates, Humans, and Future Interferon-Based Therapies

IFN-Is have been the subject of numerous interventional studies in both non-human primates and humans, aiming to harness their antiviral properties or mitigate their chronic immune-activating effects. These studies underscore the dual role of IFN-Is as both beneficial and potentially detrimental in the context of HIV infection. By targeting the IFN-I pathway through either agonists or antagonists, researchers identified promising therapeutic strategies, both as standalone interventions and also in combination with other immunomodulatory approaches.

### 6.2. IFN-I Agonists in Non-Human Primates and Humans

Agonists of IFN-Is, such as IFN-α, have shown potential in enhancing antiviral immunity. Studies in SIV-infected non-human primates, a model for HIV infection, provided significant insights. Here, early therapeutic administration of IFN-α in SIV-infected macaques demonstrated reduced viral replication and delayed disease progression [122,123]. These benefits are largely attributed to the induction of ISGs, which inhibit viral replication, and the enhancement of both innate and adaptive immune responses. However, prolonged exposure to IFN-α often leads to detrimental effects, including immune exhaustion, systemic inflammation, and impaired T-cell function. In SIV models, these effects mirror the immune dysregulation, and CD4+ T-cell depletion observed with untreated HIV infections [122,123].

In clinical studies, IFN-α has been evaluated as a therapeutic adjunct for HIV infection. Early-phase clinical trials highlighted its potential antiviral benefits as evidenced by reduced viral loads. However, prolonged administration of IFN-α was linked to significant side effects, such as systemic inflammation, fatigue, and immune dysregulation [124,125]. The challenge of balancing its antiviral efficacy with the immune activation it induces remains a significant obstacle for the long-term use of IFN agonists.

### 6.3. IFN-I Antagonists in Non-Human Primates and Humans

In response to the adverse effects associated with chronic IFN-I signaling, recent research focused on the development of IFN-I antagonists. These interventions aim to mitigate immune activation and promote immune recovery [25,62]. A particularly promising strategy involves blocking the IFN-I receptor (IFNAR) in SIV-infected macaques. Studies showed that transient IFNAR blockade reduces systemic immune activation, enhances CD4+ T-cell recovery, and improves the efficacy of ART [80,126]. These findings suggest that modulating the IFN-I axis can alleviate chronic inflammation and immune exhaustion associated with HIV/SIV infection.

In humans, clinical trials targeting IFNAR signaling are still in their early stages. However, preliminary data suggest that IFNAR inhibitors hold significant promise in reducing chronic inflammation and immune activation in HIV-infected individuals [28]. When combined with ART, these therapies could potentially enhance immune reconstitution and improve long-term patient outcomes.

### 6.4. Future Directions for Interferon-Based Therapies

The future of IFN-I-based therapies lies in developing strategies that balance their antiviral and immune-regulatory properties. One approach involves short-term, high-dose IFN-I therapy administered early in infection to maximize antiviral effects while minimizing chronic immune activation. Combining this strategy with ART could further improve viral suppression [8].

Selective activation of ISGs represents another innovative avenue. Targeting ISGs with antiviral functions while avoiding those associated with immune activation and exhaustion may enable more precise modulation of the IFN-I response. Advances in gene editing and targeted delivery technologies could facilitate this approach [127].

Combination therapies offer additional potential. For instance, pairing IFN-I agonists with immune checkpoint inhibitors, such as PD-1 or CTLA-4 blockade, could rejuvenate exhausted T-cells [128]. Similarly, combining IFN-I agonists with therapeutic HIV vaccines may enhance vaccine-induced immune responses, while pairing IFN-I antagonists with cytokine modulators like IL-7 or IL-15 could promote T-cell survival and proliferation [129].

Another promising strategy is integrating IFN-Is with latency reversing agents in a “shock and kill” approach to HIV therapy [130]. Here, IFN-Is could activate latent HIV reservoirs, making them susceptible to immune clearance. Finally, biomarker-guided interventions could tailor IFN-I-based therapies to individual patients, optimizing efficacy while minimizing adverse effects.

In summary, both agonists and antagonists of IFN-Is offer valuable therapeutic opportunities for managing HIV infection. By addressing the challenges of chronic immune activation and exhaustion through precision-type interventions, IFN-I-based therapies—whether used alone or in combination with other immune-modulatory approaches—hold the potential to transform HIV treatment strategies and advance progress toward a functional cure.

## 7. Conclusions

Although research studies contributed to our current understanding of IFN-I during the acute and chronic stages of HIV infections, several unanswered questions remain. For example, greater insights are required regarding the role of the different IFN-I subtypes and various ISGs and the implications of their perturbed regulation on the health and well-being of HIV patients. It is our opinion that more mechanistic studies investigating the role of immunometabolism and IFN-I signaling during an HIV infection are required to determine whether it is feasible to manipulate metabolic pathways to, e.g., allow for immune cell repolarization to phenotypes that should enhance the health of HIV patients.

## Data Availability

Not applicable.
